# Different wear in two highly cross-linked polyethylene liners in THA: wear analysis with EBRA

**DOI:** 10.1007/s00402-021-03832-0

**Published:** 2021-03-04

**Authors:** D. Dammerer, A. Keiler, D. Putzer, F. Lenze, M. Liebensteiner, M. Thaler

**Affiliations:** 1 Department of Orthopaedics and Traumatology, Medical University of Innsbruck, Anichstrasse 35, 6020 Innsbruck, Austria; 2 Department of Orthopaedics and Traumatology, Experimental Orthopaedics, Medical University of Innsbruck, Sonnenburgstrasse 16, 6020 Innsbruck, Austria; 3 Department of Orthopaedics and Sports Orthopaedics, Klinikum Rechts Der Isar, Technical University of Munich, Ismaninger Strasse 22, 81675 Munich, Germany

**Keywords:** Wear, EBRA, Cross-linked polyethylene, Cup migration

## Abstract

**Introduction:**

The purpose of this study was (1) to compare early wear rates in bedding-in periods of two highly cross-linked polyethylene liners frequently used in THA and (2) to evaluate risk factors indicating a possible higher wear rate.

**Materials and methods:**

1120 patients who received a Crossfire or a Marathon highly cross-linked (HXLPE) ultra-high-molecular-weight polyethylene liner in primary THA at our Department between 2004 and 2018 were retrospectively reviewed. Patients with (1) only alumina heads on HXLPE acetabular bearings, (2) a minimum of four radiographs per patient for EBRA analysis, (3) no osteolysis around the acetabular cup and (4) no dislocations that occurred during the study period were included.

**Results:**

A total of 328 patients (female: 183; male: 145; Marathon: 179; Crossfire: 149) fulfilled the inclusion criteria. Mean follow-up was 24 (range 7–51) months. With 0.22 (SD 0.27) mm mean total wear for the Marathon was three times greater than for the Crossfire, namely 0.07 (SD 0.14) mm. Mean cup migration during the investigated follow-up period was 0.7 (SD 0.8) mm for the Pinnacle and 0.5 mm (SD 0.7) for the Trident PSL cups.

**Conclusion:**

Initial early wear of highly cross-linked polyethylene in combination with alumina heads differs strongly between products. Long-term survivorship of these liners should be observed to determine whether early wear has an impact on aseptic loosening.

**Level of evidence:**

Level III (retrospective comparative study with prospective cohort).

## Introduction

Many long-term reports have described the success of total hip arthroplasty (THA) [1–5]. According to the current literature, cementless and cemented acetabular fixation show promising long-term results for up to 35 years [4–6]. Despite this improvement in fixation and the overall clinical success of THA, there continues to be concern regarding durability of the bearing surface in terms of wear, osteolysis and loosening [1–5]. Osteolysis secondary to polyethylene (PE) wear has been described as one of the primary reasons for late revision of THAs [7]. Thus, PE wear remains a common reason for revision surgery following THA, and different arthroplasty registers show that changing a PE liner because of wear becomes necessary in approximately 10–20% [8–12].

To reduce wear and improve the longevity of THA, highly cross-linked polyethylene liners were introduced for clinical use in 1998 and emerged as an alternative bearing [13]. In vitro studies suggested that conventional polyethylene can be highly cross-linked during the manufacturing process to provide a three-dimensional structure that is intrinsically resistant to wear [14–16]. During the first six months of clinical use a so-called creep deformation of the PE is mentioned rather than a wearing away of material [17]. According to the literature, in midterm studies of the highly cross-linked polyethylenes (with mean follow-up durations of approximately 5 years) investigators typically excluded the penetration data from the early bedding-in period (i.e., when creep is substantial) to obtain a more accurate measure of the actual steady-state wear rates [7]. Although during the first few years of clinical use the apparent wear rate of the highly cross-linked polyethylenes has been lower than that of traditional polyethylenes, total penetration during the first one to two years of use tends to be comparable for the two types of polyethylene, even if one wears substantially less than the other [7].

According to the Australian Orthopaedic Association National Joint Replacement Registry and its 2019 Annual Report, with a total of 8450 implanted cups the Trident PSL was the cup most commonly used in primary THA in 2018, followed by 6333 Pinnacle acetabular cups, thus making it the second most frequently used cup in primary THA in Australia [18]. During the same time, a total of 33,386 Trident PSL cups and 150,407 Pinnacle acetabular cups were implanted in THA in England, Wales, Northern Ireland and the Isle of Man. Thus, the Pinnacle was the most commonly used hip cup in England, Wales, Northern Ireland and the Isle of Man and the Trident was the second most widely used brand in 2018 [19]. In Germany, 1875 Trident and 17,878 Pinnacle cups were implanted in 2018 [20].

Overall, at 18 years the cumulative percent revision of total conventional hip replacement with XLPE is 7.2%; but to date there is no report about the two in our study investigated liners [18].

The purpose of this study was (1) to compare early wear rates in bending-in periods of two frequently used highly cross-linked polyethylene liners in THA and (2) to evaluate risk factors indicating a possible higher wear rate.

## Materials and methods

The study was approved by the local ethics committee. Written informed consent was obtained from all subjects before participation. All methods and measurements were carried out in accordance with relevant guidelines and regulations.

**Fig. 1:**
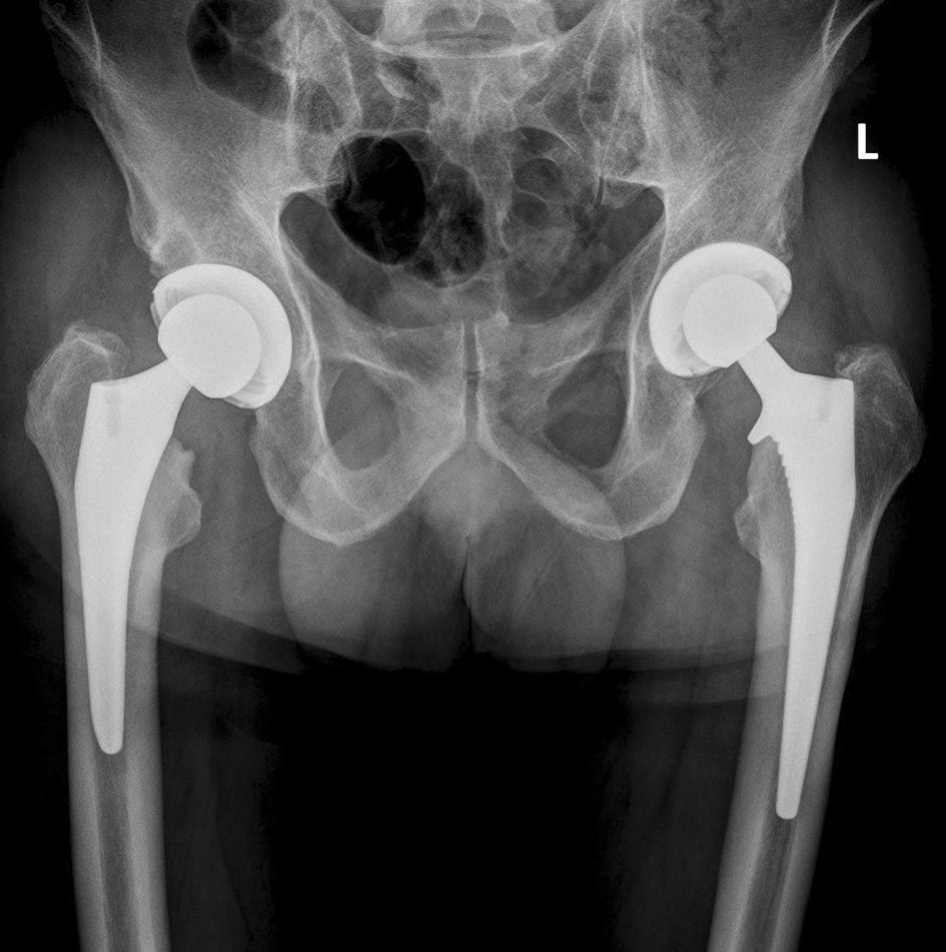
Example of a patient after bilateral THA that received a Marathon liner in a Pinnacle cup (left hip) and a Crossfire liner in a Trident PSL cup (right hip)

All consecutive patients who received a Crossfire (Stryker Orthopaedics, Mahwah, NJ, USA) or a Marathon (DePuy Synthes, Warsaw, IN, USA) highly cross-linked (HXLPE) ultra-high-molecular-weight polyethylene (UHMWPE) liner in a primary THA between 2004 and 2018 at our Department were retrospectively reviewed. During this time, a total of 1120 (Crossfire *n* = 636; Marathon *n* = 484) of the above-mentioned PE liners were implanted as acetabular bearing surface in primary THA. All Crossfire liners were used in Trident PSL cups (Stryker Orthopaedics, Mahwah, NJ, USA) and all Marathon liners were used in Pinnacle cups (DePuy Synthes, Warsaw, IN, USA). Figure 1 gives an example of a patient after bilateral THA that received both implants. The decision for one or the other type of cup was made by the surgeon himself, who preferred to use a particular cup type, and was not made by the patient. Thus, there was no selection bias.

Included were (1) only alumina heads on HXLPE acetabular bearings, (2) a minimum of four radiographs per patient for EBRA analysis, (3) no osteolyses were found around the acetabular cup and (4) no dislocation of the THA occurred during the investigated time period. Exclusion criteria were (1) revision of either the acetabular or (2) the femoral component, (3) a diagnosis of infection.

Prosthetic stability, PE wear and cup migration were assessed retrospectively with EBRA (German: Einzel-Bild-Röntgen-Analyse) [21] from plain x rays. EBRA is a well-established method that evaluates standard anterior–posterior radiographs without requiring additional means at exposure (e.g., ball markers). Simulating the spatial situation, it computes parameters of longitudinal and transverse migration of prosthetic cup, femoral head and wear. The migration of the femoral head, the acetabular cup and the wear in the horizontal and vertical directions can be studied. Total wear was calculated from the EBRA wear results in the horizontal and vertical directions by vectoral addition to make the results comparable with the results of other methods. Furthermore, total wear was calculated as the difference between migration of the head and cup in the horizontal and vertical directions. A comparability algorithm using a grid of transverse and longitudinal tangents of the pelvis contour divides serial radiographs into sets of comparable ones. Migration is measured only between comparable radiographs [21]. According to Callary et al., the variability in wear measurements observed with EBRA as compared to RSA is similar when using different acetabular reference segments [22].

In our Department, patients are followed with radiographs routinely before discharge, 6 weeks after surgery and 12 months postoperative. Additional radiographs are performed if the patient has any complaints with the THA. All radiographs were taken with the same technique (anterior–posterior (AP) radiographs; patient standing in upright position and full weight-bearing) following the EBRA protocol. For EBRA investigation, a minimum of four radiographs per patient and a minimum radiological follow-up of six months were required for this analysis. Wear and cup migration analysis was conducted with EBRA by one independent investigator, who was not involved in the surgeries or postoperative treatment of the patients.

The Marathon is a moderately cross-linked UHMWPE (ultra-high-molecular-weight polyethylene) liner that was clinically introduced in 1998 [23]. The liner is currently available for the Pinnacle and the Duraloc (DePuy Synthes, Warsaw, IN, USA) acetabular component system. In the Marathon process, an extruded rod bar stock is irradiated with a dose of 50 kGy and then remelted at 150° [24]. After remelting, the rods are then annealed at 120° for 24 h [25]. Acetabular components are machined from the processed bar stock, enclosed in gas-permeable packaging, and then gas plasma-sterilized [26].

The Crossfire is an annealed highly cross-linked UHMWPE liner and was clinically introduced in 1998. The liner is currently available for the Trident acetabular cup design. In the Crossfire process, an extruded rod bar stock is irradiated with a nominal dose of 75 kGy and then annealed at 130° [27]. Acetabular components are machined from the bar stock, barrier-packaged in nitrogen (N2-VAC) and then gamma-sterilized with a nominal dose of 30 kGy. Consequently, components that have been through the Crossfire process have received a total dose of 105 kGy [26, 28].

### Statistical analysis

Mean, median, range, and standard deviation were calculated for the different measurement parameters. Migration and wear rate were calculated as vectors of their corresponding components. For the analysis, Access and Excel (Microsoft Office Professional Plus 2010, Redmond, WA, USA) as well as Graph Pad Prism (Version 8.0, GraphPad Software, Inc., La Jolla, CA, USA) were used. The Kolmogorov–Smirnov test was performed to assess normal distribution of data. The independent samples Mann–Whitney U test was used to assess statistical significance in migration, wear rates, inclination and anteversion. A *p* value of 0.05 was considered statistically significant.

## Results

A total of 328 patients (female: 183; male: 145) fulfilled the inclusion criteria. For the Pinnacle study group a total of 179 patients (female: 100; male: 7) were recruited. Mean patient age at surgery was 69 (range 18–90) years and mean body mass index was 26.9 (range 18–50) kg/m^2^. Mean follow-up was 24 (range 7–51) months. For the Trident PSL group 149 patients (female: 83, male: 66) were recruited. At the time of surgery, mean patient age was 65 (range 33–89) years and mean body mass index was 26.8 (range 15–39) kg/m^2^. Mean follow-up was four (range 2–9) years. Mean cut-to-suture time was 78 (range 36–209) min for the Pinnacle group and 72 (range 33–188) min for the Trident PSL group. In both study groups, a direct anterior approach [29] was used in supine position in almost all surgeries (Pinnacle: 99%; Trident PSL: 99%). Three patients (Pinnacle: 1%; Trident PSL: 1%) were operated using a transgluteal approach [30]. The preoperative diagnosis was osteoarthritis in over 80% of both patient cohorts, followed by avascular necrosis of the femoral head (Pinnacle: 12%; Trident PSL: 6%) and femoral neck fracture (Pinnacle 2%; Trident PSL: 1%). In the Pinnacle group nine (6%) patients were preoperatively diagnosed with dysplastic hip deformation. Details are shown in Table 1.

**Table 1:** Patient demographics (mean) of the study group. Range is given in brackets

	Pinnacle (%)	Trident PSL (%)
Number of patients		
Female	100 (56%)	83 (56%)
Male	79 (44%)	66 (44%)
Total	179	149
Mean age (years)	67 (18–90)	64 (32–89)
BMI (kg/m^2^)	26.8 (18.3–50.8)	26.8 (15.2–39.4)
Cut-to-suture time (min)	78 (36–209)	72 (33–188)
Surgical approach		
Direct anterior approach	177 (99%)	146 (99%)
Transgluteal approach	2 (1%)	1 (1%)
Surgical position		
Supine	179 (100%)	149 (100%)
Preoperative diagnosis		
Osteoarthritis	153 (85%)	130 (87%)
Avascular necrosis of the femoral head	22 (12%)	9 (6%)
Fractures of femoral neck	3 (2%)	1 (1%)
Dysplastic hip	0 (0%)	9 (6%)
Pathologic FX	1 (1%)	0 (0%)

**Fig. 2:**
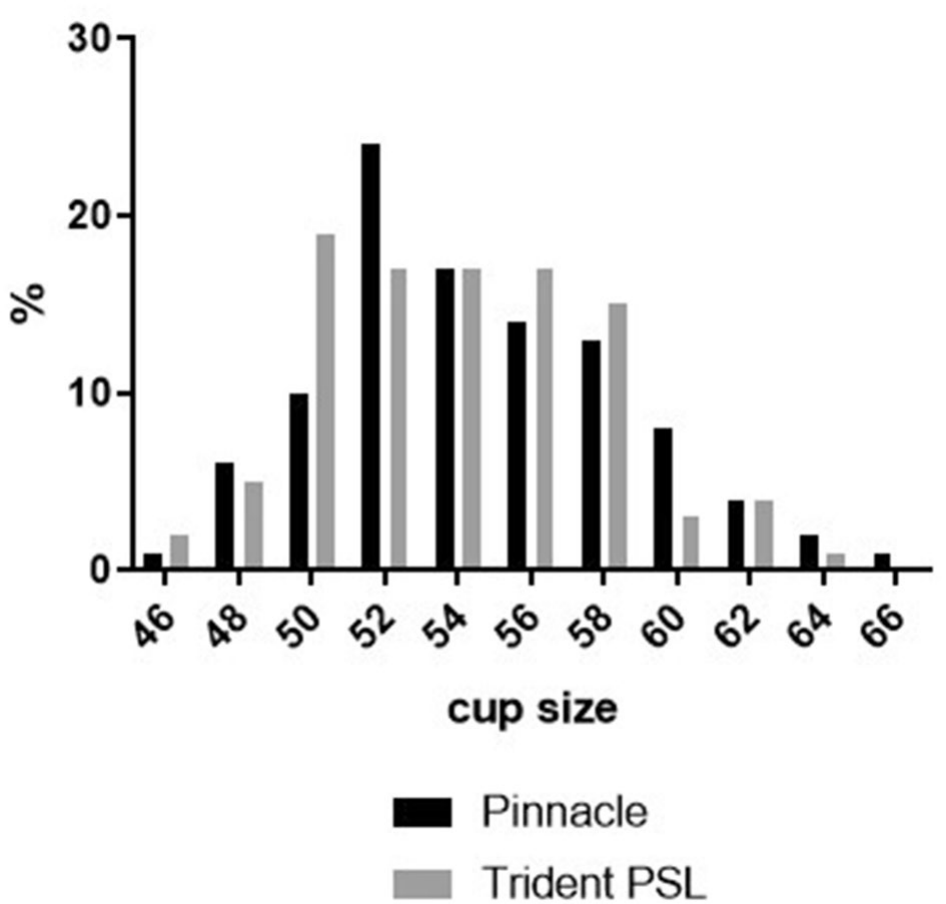
Implanted cup sizes for the Pinnacle and the Trident PSL cohort

In all investigated cases alumina heads on HXLPE acetabular bearings were used. The most commonly used head size in both groups was 32 mm (*n* = 163 for the Pinnacle and *n* = 100 for the Trident PSL study group). Other head sizes used were 28 mm (*n* = 12 for the Pinnacle and *n* = 20 for the Trident PSL group) and 36 mm (*n* = 2 for the Pinnacle and *n* = 19 for the Trident PSL group). Figure 2 shows the implanted cup sizes. For the Pinnacle group, cup size ranged from 46 to 66 mm, for the Trident PSL group size ranged from 46 to 64 mm.

**Fig. 3:**
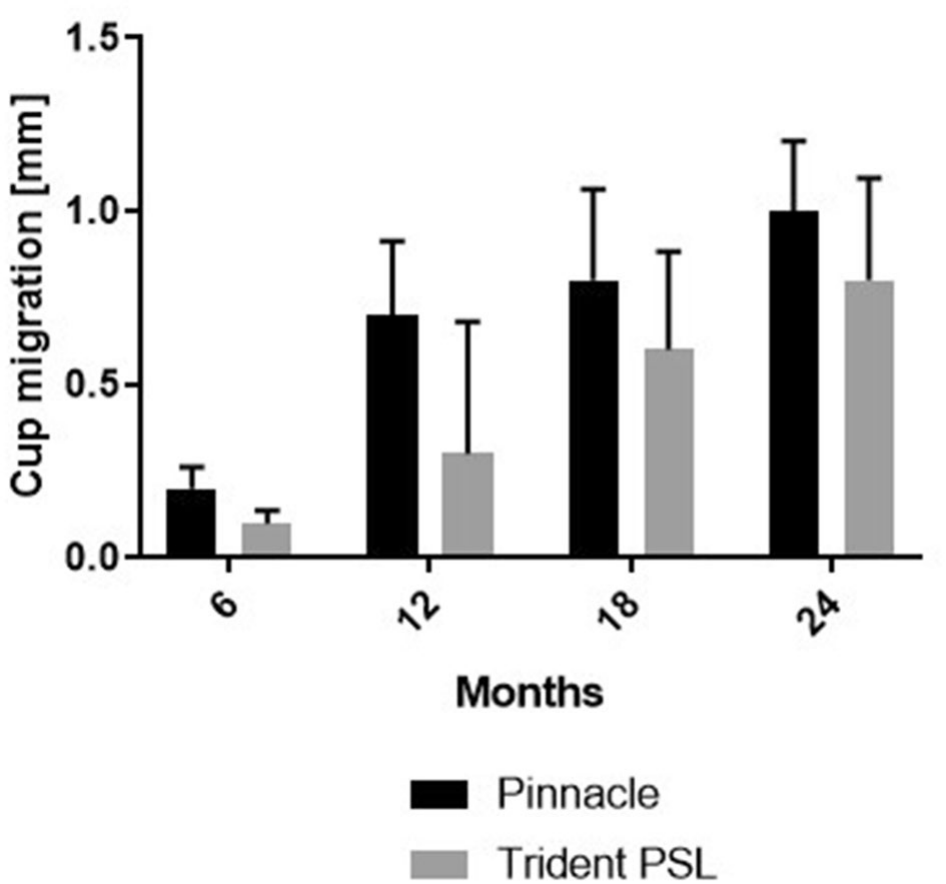
Median cup migration for Trident PSL and Pinnacle. Bars indicate the 95% confidence interval

According to the Kolmogorov–Smirnov test, migration, inclination, anteversion and wear rate were not normally distributed in the two groups (*p* < 0.05). Median cup migration during the investigated follow-up period was 0.5 mm (95% confidence interval; 0.6–0.8) for the Pinnacle and 0.2 mm (95% confidence interval; 0.3–0.5) for the Trident PSL cups (Fig. 3). Cup migration was statistically significantly greater for the Pinnacle group than for the PSL group at 6 months (*p* = 0.023), 12 months (*p* = 0.005) and 18 months (*p* = 0.038) follow-up. No statistically significant difference was found between the two groups after 24 months follow-up time (*p* = 0.089).

**Fig. 4:**
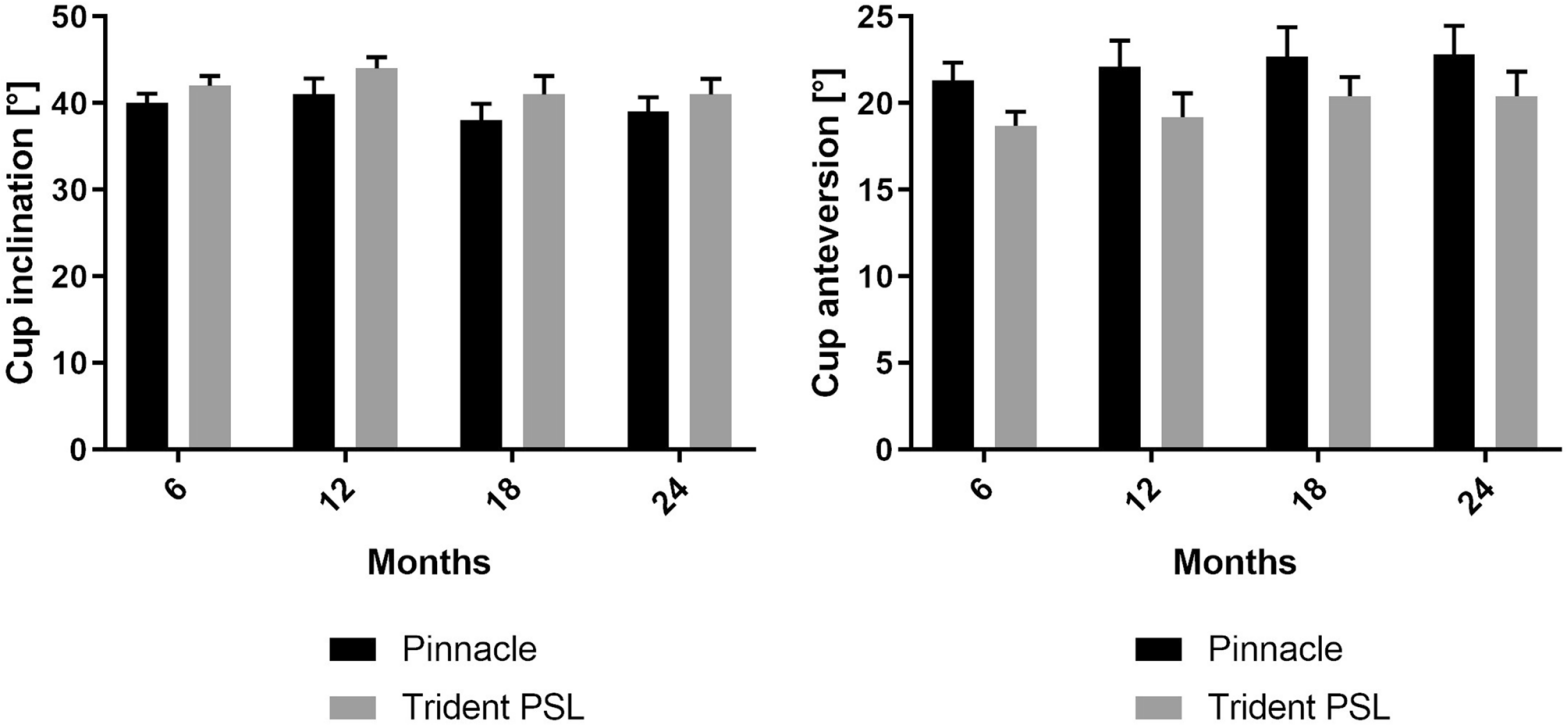
Median cup inclination and anteversion (bars indicate the 95% interval) for the clinical follow-up period of two years for the two study groups under investigation. Bars indicate the 95% confidence interval (Pinnacle and Trident PSL)

Cup inclination was 2° greater for Trident PSL cups than for Pinnacle cups at six months follow-up (*p* = 0.003). No statistically significant difference was found between the two groups at 12 months (*p* = 0.069), 18 months (*p* = 0.082) or 24 months (*p* = 0.081) follow-up time (Fig. 4, Table 2).

**Table 2:** Details on cup median migration, inclination, anteversion and wear rate

	Cup type	6 months	12 months	18 months	24 months
Median inclination (°)	Trident PSL	42 (41–44)	44 (41–46)	41 (40–44)	41 (39–44)
	Pinnacle	40 (39–41)	41 (39–43)	38 (38–42)	39 (37–41)
Median anteversion (°)	Trident PSL	19 (18–20)	19 (18–20)	21 (19–21)	20 (19–22)
	Pinnacle	21 (20–22)	22 (21–24)	22 (21–24)	23 (21–24)
Median migration (mm)	Trident PSL	0.1 (0.1–0.2)	0.3 (0.3–1.0)	0.6 (0.5–1.0)	0.8 (0.7–1.2)
	Pinnacle	0.2 (0.2–0.3)	0.7 (0.7–1.1)	0.8 (0.8–1.4)	1.0 (0.9–1.4)
Median wear (mm)	Trident PSL (Crossfire)	0.00 (0.03–0.06)	0.10 (0.08–0.14)	0.14 (0.14–0.23)	0.14 (0.17–0.29)
	Pinnacle (Marathon)	0.05 (0.05–0.08)	0.14 (0.15–0.26)	0.22 (0.19–0.34)	0.28 (0.27–0.46)

95% confidence interval is given in brackets. All values in degrees

Pinnacle cups showed greater cup anteversion than did the PSL group (Fig. 4, Table 2). Pinnacle cups showed a statistically significantly greater anteversion of 2° measured after six months (*p* < 0.001), 3° greater anteversion measured after 12 months (*p* = 0.012), 1° greater anteversion measured after 18 months (*p* = 0.048) and 3° greater anteversion (*p* = 0.026) at the end of the follow-up period (24 months).

**Fig. 5:**
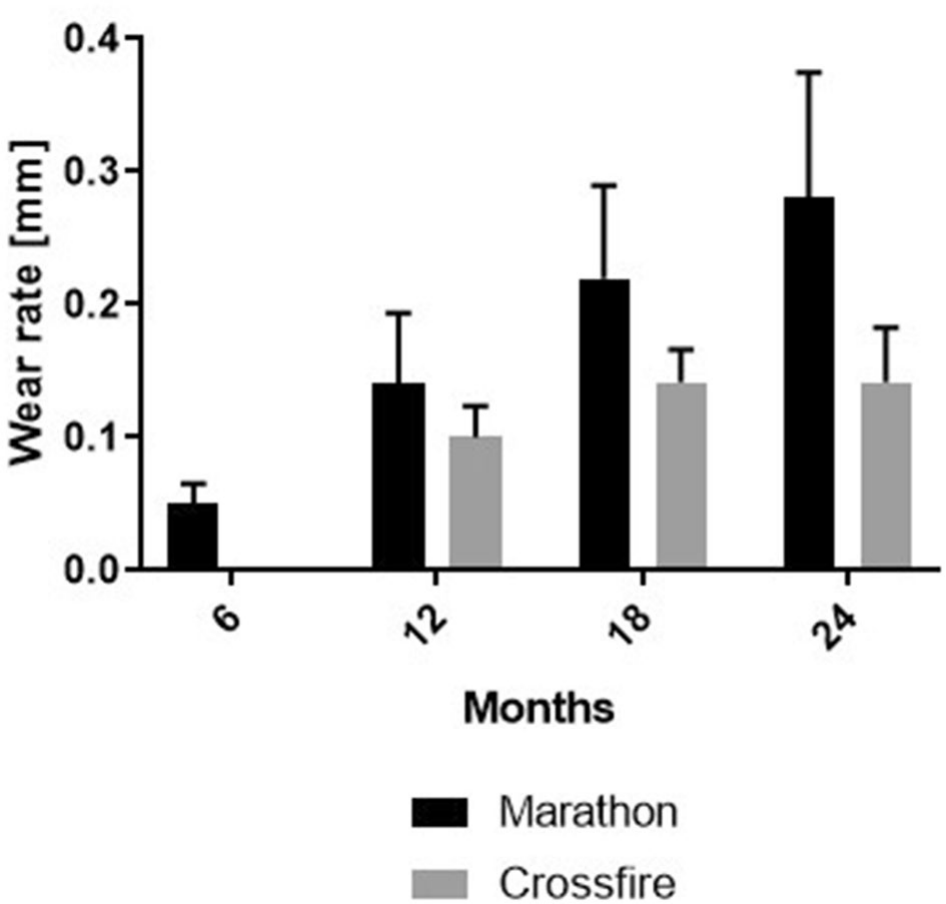
Median wear rate of the two liners under investigation (Marathon—Pinnacle, Crossfire—Trident PSL) for the clinical follow-up period of two years. Bars indicate the 95% confidence interval

The wear rate showed a statistically highly significant difference between the two liners after 12 (*p* < 0.0001), 18 (*p* = 0.018) and 24 (*p* = 0.002) months (Fig. 5, Table 2). The Marathon liner showed a 0.4 mm higher wear rate after 12 months, a 0.8 mm higher wear rate after 18 months and a two-fold higher wear rate after 24 months than did the Crossfire. No statistical difference was found between the two liners after six months of our observed time period (*p* = 0.261) (Fig. 5, Table 2).

Median total wear was 0.11 (95% interquartile interval; 0.15–0.21) mm for the Marathon and 0.10 (95% Interquartile interval; 0.10–0.13) mm for the Crossfire liner.

## Discussion

The presented study investigated early wear in two very frequently used highly cross-linked UHMWPE acetabular liners in primary THA. The most important findings of the study were that the Marathon liner had a significantly higher wear rate than did the Crossfire and that the Pinnacle cup showed a higher migration rate than did the Trident PSL cup in the first two years postoperative. Additionally, to the best of our knowledge, this study presents the largest patient cohort to date, with more than 100 participants in each study group.

Previously published studies investigating the bearing surface in primary THA showed that wear and cup migration in the first two years postoperative are good predictors of later THA failure [31, 32]. The literature reports long-term wear rates of 0.04 ± 0.02 mm/year for highly cross-linked polyethylene liners versus wear rates of 0.08 ± 0.03 mm/year for conventional polyethylene [33]. These initial findings are not only useful for describing the mechanical properties of different PE liners, but are also of special clinical interest as a significant correlation was demonstrated between wear and reduced revision due to aseptic loosening and osteolysis [31, 34]. In mid- and long-term studies of HXLPE, investigators typically excluded the penetration data from the early bedding-in period (e.g., when creep is substantial) [7, 17]. This was done to obtain a more accurate measure of the actual steady-state wear rates and to exclude the creep deformation of PE, which typically occurs in the first six months of clinical use [7, 17]. The literature shows that investigations of the yearly wear rate of Marathon and Crossfire liners revealed a mean wear rate for the Marathon ranging from 0.01 to 0.08 (± 0.07 to ± 0.24) mm/year, which is similar to the yearly wear rate of the Crossfire, namely ranging from 0.01 to 0.12 ± 0.05 mm/year [35–41]. Tables 3 and 4 give an overview of peer-reviewed studies involving Crossfire (Table 3) and Marathon (Table 4) liners.

**Table 3:** Summary of peer-reviewed studies involving Crossfire liners (adopted from UHMWPE Biomaterials Handbook: Ultra High Molecular Weight Polyethylene in Total Joint Replacement and Medical Devices 2nd edition, Steven M. Kurtz, 2009 [46] and from UHMWPE Biomaterials Handbook: Ultra High Molecular Weight Polyethylene in Total Joint Replacement and Medical Devices 3rd edition, Steven M. Kurtz and William Andrew, 2015 [47])

Crossfire	Martell 2003 [41]	Röhrl 2005 [40]	Krushell 2005 [39]	D’Antonio 2005 [48]	Röhrl 2007 [38]	Rajadhyaksha 2009 [49]	Capello 2011 [50]	Röhrl 2012 [51]	Epinette 2014 [52]
Study type	RCT	Pcoh	Hcoh	Hcoh	Pcoh	Hcoh	Hcoh	Pcoh	Hcoh
Cup design	Secur-Fit HA PSL	Osteonics	Microstructured PSL	Microstructured PSL	Osteonics	Microstructured PSL	Secur-Fit HA PSL	Osteonics	Trident Arc2f threaded cup
Cup fixation	Uncemented	Cemented	Uncemented	Uncemented	Cemented	Uncemented	Uncemented	Cemented	Uncemented
Head size	28 mm	28 mm	28 mm	28 mm	28 mm	28 mm	28 mm	28 mm	28 mm or 32 mm
Head material	CoCr L-Fit	CoCr	CoCr L-Fit	CoCr L-Fit	CoCr	CoCr L-Fit	CoCr	CoCr	ceramic
N (Cross-fire)	24	10	40	56	10	27	42	8	228
Mean follow-up in years	2.3	3.0	4.0	4.9	6.0	5.9	8.6	10.0	10.5
2-D liner penetration (mm/y)	0.12 ± 0.05	0.02	0.05 ± 0.02	0.06 ± 0.02	0.01	0.05 ± 0.04	0.03 ± 0.01	0.02	0.02 ± 0.01

*Hcoh* retrospective cohort study (Level III), *Pcoh* prospective cohort study, *RCT* randomized controlled trial (Level I), *L-Fit* low friction ion treatment; 2-D linear wear is listed for the longest follow-up period and includes the initial bedding-in period

**Table 4:** Summary of peer-reviewed studies involving Marathon liners (adopted from UHMWPE Biomaterials Handbook: Ultra High Molecular Weight Polyethylene in Total Joint Replacement and Medical Devices 2nd edition, Steven M. Kurtz, 2009 [46] and from UHMWPE Biomaterials Handbook: Ultra High Molecular Weight Polyethylene in Total Joint Replacement and Medical Devices 3rd edition, Steven M. Kurtz and William Andrew, 2015 [47])

Marathon	Hopper 2003 [35]	Heisel 2004 [36]	Heisel 2005 [53]	Engh 2006 [37]	Bitsch 2008 [43]	Engh 2012 [54]	Bedard 2014 [1]
Study type	Pcoh	Pcoh	Pcoh	RCT	Hcoh	RCT	Hcoh
Cup design	Duraloc 100	Duraloc or Pinnacle	Duraloc	Duraloc 100	Duraloc or Pinnacle	Duraloc 100	Pinnacle
Cup fixation	Uncemented	Uncemented	Uncemented	Uncemented	Uncemented	Uncemented	Uncemented
Head size	28 mm	28 or 32 mm	28 mm	28 mm	28 or 32 mm	28 mm	28 or 32 mm
Head material	CoCr	CoCr or ceramic	CoCr	CoCr	CoCr or ceramic	CoCr	CoCr
N (Marathon)	48	34	3	116	32	116	106
Mean follow-up in years	2.9	2.8	3.2	5.7	5.8	10.6	8.9
2-D liner penetration (mm/y)	0.08 ± 0.24	0.02 ± 0.1	0.06 ± 0.02	0.01 ± 0.07	0.03 ± 0.04	0.04 ± 0.06	0.05 ± 0.16

*Hcoh* retrospective cohort study (Level III), *Pcoh* prospective cohort study, *RCT* randomized controlled trial (Level I), *L-Fit* low friction ion treatment; 2-D linear wear is listed for the longest follow-up period and includes the initial bedding-in period

Whereas reported data show similar wear rates for the Crossfire group, there is a discrepancy in the wear rates for the Marathon liner. In our study, we found a highly significant difference in mean wear rate of 0.20 mm (SD 0.21) for the Marathon in comparison to 0.09 mm (SD 0.13) for the Crossfire (*p* < 0.0002). Both liners are XLPE, but there are differences in production, as mentioned in the Material and Methods section. According to the Australian Orthopaedic Association National Joint Replacement Registry (AOANJRR) and its 2019 Annual Report, XLPE is classified as ultra-high-molecular-weight polyethylene that has been irradiated with high-dose (≥ 50 kGy) gamma or electron beam radiation [18]. The Marathon is irradiated with a dose of 50 kGy, whereas the Crossfire is irradiated with a nominal dose of 75 kGy and then gamma-sterilized with a nominal dose of 30 kGy. Consequently, components that went through the Crossfire process received a total dose of 105 kGy [24, 27, 28]. The Crossfire received twice the dose of irradiation that the Marathon liner received. Therefore, the Marathon is stated to be a moderately cross-linked liner [23] compared to the highly cross-linked Crossfire. Our results suggest that the differences in the production of XLPE liners have a strong impact on their early wear rate.

It is well known that the size and material of the femoral head are further parameters influencing wear. Patients in both of our study groups received exclusively alumina ceramic heads. The majority of femoral head sizes implanted in our study was 32 mm and the current literature, for XLPE, shows the 32-mm head size to have the lowest rate of revision [18]. In mid- to long-term studies mainly 28 mm femoral heads were used. A recently published study by Gaudin et al. found no significant difference in either the mean linear or volumetric wear rates for 32 mm or 36 mm ceramic heads and highly cross-linked polyethylene [42]. This is also well in line with the 2019 Annual Report of the AOANJRR [18].

In clinical practice, increased liner wear rate does not only go along with an increased risk of osteolysis, loosening and revision rate [7]. There is a high rate of THA dislocation and a high rate of cup revision after liner exchange, as could be described in a recent publication by Dammerer et al. [55]. This means that every sixth patient with isolated liner exchange can expect to experience dislocation due to wear [55]. Therefore, we assume that our findings are not only statistically significant, but clinically relevant.

This study has several limitations including the retrospective methodology. Secondly, the follow-up period is short, which is certainly the study’s most important limitation. Although there are some studies with a longer follow-up period, in most cases the number of patients included is much smaller than in our study. Moreover, to our knowledge, no other study describing the wear rates of Marathon and Crossfire liners has a sample size comparable to that of our study. Third, we used EBRA to measure cup migration and the wear rate of the PE liners. The radiographic assessment of small amounts of wear by means of the method used in the study poses some limitations, which have been recognized but are not unique to our study [43–45]. Additionally, the method of radiographic assessment of the wear rate on two-dimensional plane x ray pictures does not permit a volumetric wear rate to be calculated. We present only the two-dimensional linear penetration in millimeters. However, EBRA facilitates accurate and reliable analysis of wear and migration of components in hip arthroplasty [42]. Nevertheless, we used the same method to measure both components, the Marathon and the Crossfire liner. Consequently, we consider our data and results to be reliable, reproducible and comparable with the existing literature.

## Conclusion

The Marathon liner had a significantly higher wear rate than the Crossfire liner and the Pinnacle cup showed a higher migration rate than the Trident PSL cup in the first 2 years postoperative. As the impact of our findings on long-term survival is not known, we recommend that long-term survival of both liners and cups investigated in the current study should be observed. In conclusion, we suggest that surgeons should be informed and aware of the different wear rates of two common highly cross-linked polyethylenes in combination with alumina heads.
